# First isolation and characterization of a bovine parainfluenza virus type 3 genotype C strain from an aborted Holstein fetus

**DOI:** 10.3389/fvets.2025.1598013

**Published:** 2025-06-05

**Authors:** Yunxin Ren, Xi Chen, Cheng Tang, Hua Yue

**Affiliations:** College of Animal and Veterinary Sciences, Southwest Minzu University, Chengdu, China

**Keywords:** BPIV3, abortion, isolation and characterization, phylogenetic analysis, genomic characterization

## Abstract

Bovine parainfluenza virus type 3 (BPIV3) is a significant pathogen responsible for bovine respiratory disease complex (BRDC) and reproductive disorders. Despite its importance, genomic studies of BPIV3 in abortion samples are currently lacking. We detected a BPIV3-positive sample via PCR in the lung sample from an aborted Holstein fetus; therefore, this study aimed to isolate and identify a BPIV3 strain from the aborted fetus sample of Holstein and analyze its genome. We successfully isolated and obtained a BPIV3 strain using MDBK cell lines, named LC1. Phylogenetic analysis based on complete genome sequences revealed that the LC1 strain belongs to genotype C and clusters into a branch with the Chinese strain SD2020. Notably, unique amino acid mutations were identified in the P (I297T) and M (P5N) proteins of the LC1 strain. To the best of our knowledge, this is the first BPIV3 genotype C strain to be isolated from a bovine abortion sample, as well as the first characterization of the abortion source BPIV3 genome reported. This finding lays the foundation for further studies on the genetic diversity of BPIV3.

## Introduction

Bovine parainfluenza virus type 3 (BPIV3), a member of the *Respirovirus* genus in the family *Paramyxoviridae*, is one of the most critical viruses causing bovine respiratory disease complex (BRDC), which results in significant economic losses to the global cattle industry (1, 2). BPIV3 primarily infects cattle, damaging the lower respiratory tract, triggering interstitial pneumonia, and leading to immunosuppression of the host organism (3). In addition, BPIV3 is associated with abortion in dams and has attracted increasing attention for its role in the reproductive health (4, 5). BPIV3 was first detected in BRDC cases in 1959 (6). BPIV3 is an enveloped, non-segmented, negative-strand RNA virus with a genome approximately 15 kb in size. It encodes six structural proteins (N, P, L, M, HN, and F) and three non-structural proteins (C, V, and D) (7–9). Currently, BPIV3 is classified into three genotypes (A, B, and C) based on phylogenetic analyses (10, 11), and it has been characterized as having a worldwide distribution (12–15).

BPIV3 was isolated from aborted fetal cows in Ohio as early as 1965 (16). Subsequent isolations of BPIV3 from aborted fetal cows have been reported in Portugal and Brazil, but the genome and genotype of these strains have not been reported (16, 17). Research data on BPIV3 related to abortion in pregnant cattle remain scarce. One study showed that natural infection with BPIV3 genotype A in a Holstein cattle resulted in abortion, with the lungs of the aborted fetus exhibiting diffuse reddening and a rubbery texture (5). Additionally, several experimental studies of in-utero infections have found that BPIV3 was able to cause abortions in two out of six dams and that surviving calves were weak, unable to stand after birth, and developed interstitial pneumonia (17, 18). However, the precise mechanism of transmission remains unclear, particularly whether BPIV3 can cross the placental barrier to directly infect the fetus following maternal infection. A study conducted in Argentina provided new insights by detecting BPIV3 genotype B in the vaginal tissues of infected dams, suggesting a potential transmission route (13).

Despite the long-standing recognition of BPIV3’s association with miscarriages, there remains a scarcity of genomic studies exploring the pathogenesis and transmission of its strains. In particular, there is a significant gap in global genomic sequence data concerning BPIV3 derived from aborted samples, which limits our comprehensive understanding of its molecular characteristics. Therefore, this study aims to fill this gap by isolating and characterizing BPIV3 from aborted fetus, and conducting genomic analysis. By elucidating the genome sequences of these strains, we aim to uncover the genetic evolutionary characterization of BPIV3, thereby enhancing our understanding of the molecular mechanisms underlying BPIV3-induced abortions.

## Materials and methods

### Sample collection and processing

The abortion case occurred in April 2022 on a Brucella abortion-free dairy farm in Heilongjiang, China. A primiparous cow aborted on the 7th day after being moved to a new pen by the farmer, at 240 days of gestation. The aborted fetus was observed cosmetically and was complete in appearance, with no obvious tissue loss or breakage, and body parts were well-proportioned, with no obvious developmental malformations. Sterile samples were collected from the heart, liver, spleen, lungs, kidneys, and inguinal lymph nodes of the aborted fetus. The tissues were processed using a Tissuelyser-24 grinder (Jingxin, Shanghai, China), and DNA and RNA were extracted from each sample using a DNA kit (Omega Bio, New York, United States) and RNAios Plus (TaKaRa Bio, Inc., Kusatsu, Japan), respectively. The RNA was then reverse-transcribed into cDNA using the PrimeScript^™^ RT reagent commercial kit (TaKaRa) and stored at −20°C for further analysis. Screening for common reproductive disorder pathogens, including bovine viral diarrhea virus, bovine herpesvirus type 1, *Mycoplasma bovis*, Leptospira, and BPIV3, was performed according to reported PCR or RT-PCR methods (19, 20). The primer sequences used for BPIV3 detection were as follows: forward primer, 5′-GAATGACTCATGATAGAGGTAT-3′; reverse primer, 5′-AGGACAACCAGTTGTATTACAT-3′.

### Virus isolation and identification

To isolate the virus, 1 mL of PBS was added to homogenized samples and centrifuged at 10,000 rpm for 15 min at 4°C. The supernatant was then collected and filtered through a 0.22 μm filter before inoculation into MDBK cells cultured in a medium containing 2% fetal bovine serum. MDBK cells were maintained at 37°C and 5% CO_2_. Blind passages were conducted every 7 days, and after three rounds of blind passages, the isolate was analyzed using RT-PCR. Subsequently, the isolate was purified and subjected to TCID_50_ assay, indirect immunofluorescence assay, transmission electron microscopy, and hemagglutination (HA) test using 1% chicken red blood cells, based on our previous studies (20).

### Genome amplification and analysis

Eight primer pairs were designed based on the known BPIV3 genotype C genome (GenBank accession number KT071671) to amplify the BPIV3 genome from BPIV3-positive lung tissue sample. The primer sequences are shown in Table 1. The PCR products were purified and cloned into the pClone007-T Simple Vector and sequenced by Tsingke Biotechnology Co., Ltd. Sequence assembly and multiple sequence similarity analyses were performed using the SeqMan and MegAlign program of DNASTAR 7.0 software (DNASTAR Inc., Madison, WI, United States). Multiple sequence alignment and phylogenetic analysis were performed with the MEGA X software to construct a maximum-likelihood phylogenetic tree. The substitution model for the constructed phylogram was selected by the software using the Find Best DNA/Protein Models (ML) option (21). Recombination events were assessed using Recombination Detection Program 4.0 (RDP 4.0, version 4.96). The protein 3D structure was predicted using the online tool ALPHAFOLD,^1^ and amino acid residues were mutated using the Mutagenesis module of PyMOL software for subsequent research.

**Table 1:** PCR primers for complete genome amplification of BPIV3.

Name	Primer sequence (5′–3′)	Position
1F	ACCAAACAAGAGGAGAGACTTG	1–1,963
1R	ATTGTTGTGCTGAGCCTTGT	
2F	AGACTCCATCCACAACCCA	1,750–3,697
2R	CTTGTGTCTGGGAACTACTGTG	
3F	TCAAAGGCAAAACAGTCATACAT	3,482–5,728
3R	TCCTACTGAGCTTTGAATTGACTGT	
4F	AACAGTACTAGTTCCAGGAAGAAGC	5,396–7,884
4R	GGACAGCCAGTTAAATTGCATATTAC	
5F	CAGTAGGACCGGGGATTTA	7,880–9,902
5R	TGTCCTCCGTGTCTTTCTCTA	
6F	CCTTTTTCCGAACTTTTGG	9,734–12,509
6R	GTAGTCACTGGTGTCAGAATCTTTA	
7F	AGGAGGAAGAATGATAAATGG	12,105–13,822
7R	TTCTTGGATTATCGTCACAGTTA	
8F	GTGTGTTGTTTAGCAGAAATAGC	13,501–15,474
8R	ACCAAACAAGAGAAAAACTCTGT	

### Selection pressure analysis

To explore the evolutionary pressures BPIV3, we employed a codon-based maximum likelihood method to calculate the ratio of non-synonymous (dN) to synonymous (dS) variations per site. A dN/dS ratio exceeding 1 indicates positive or diversifying selection, while a ratio below 1 suggests negative or purifying selection, and a ratio of 1 signifies neutral evolution. The lower bound of dN/dS values is zero. The selection pressure on genes encoding proteins with unique amino acid mutations were further analyzed using four distinct methods: fixed-effects likelihood (FEL), mixed-effects evolutionary model (MEME), single-likelihood ancestor counting (SLAC), and Fast Unconstrained Bayesian AppRoximation (FUBAR). These analyses were conducted via the online tool Datamonkey.^2^ All methods consistently identified specific loci under positive selection, underscoring their evolutionary significance.

## Results

**Figure 1:**
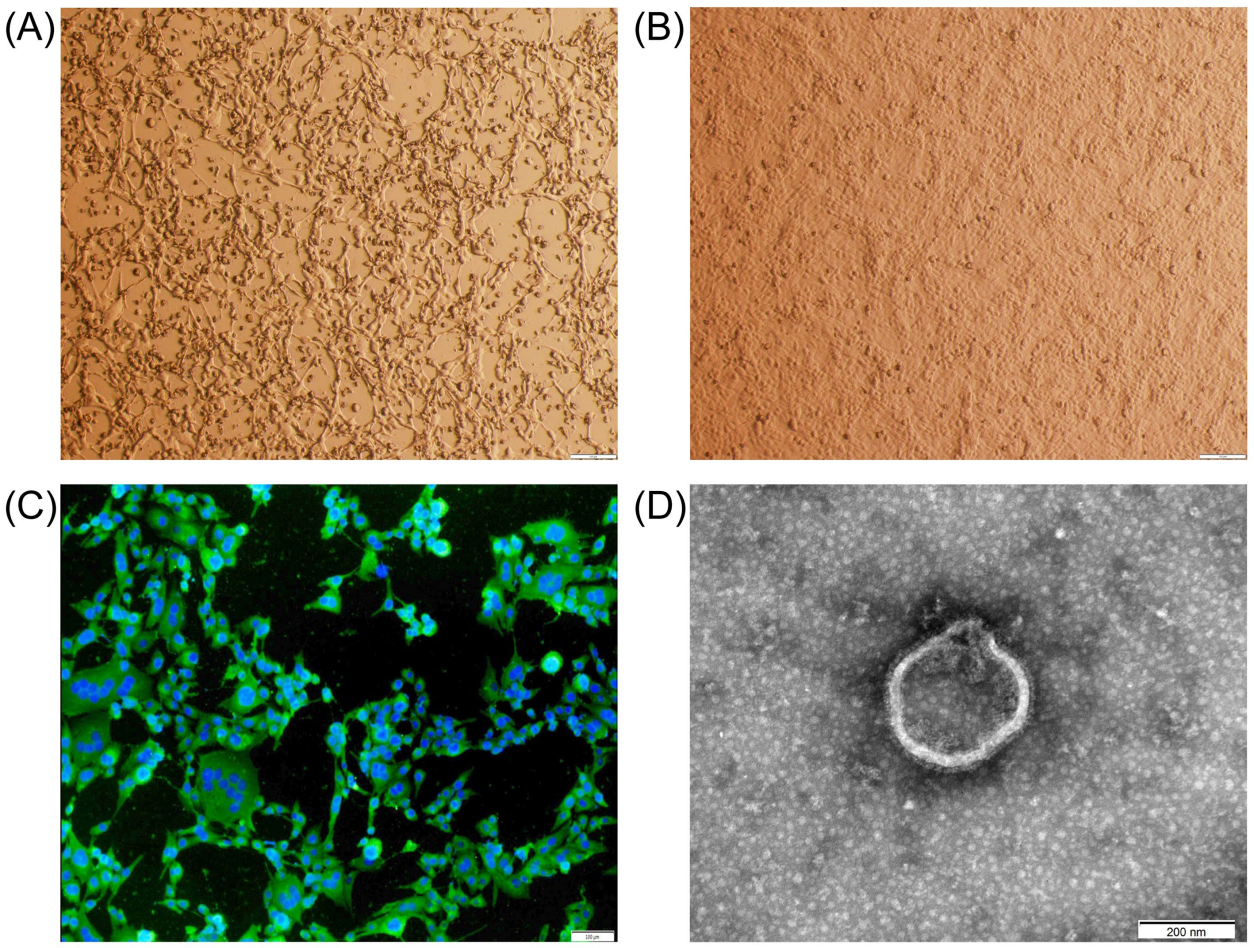
Microscopic examination of the abortion-derived BPIV3. **(A)** CPE in MDBK cells at 48 h post-inoculation (shrinkage, shedding, and fusion). **(B)** Negative control MDBK cells after 48 h of culture. **(C)** Immunofluorescence assay of the isolate in MDBK cells (×40). The MDBK cells were incubated with a rabbit anti-BPIV3 whole-virus polyclonal antibody, followed by FITC-conjugated goat anti-rabbit IgG. **(D)** Transmission electron micrograph of the isolate (×40,000).

Out of six samples, BPIV3 positivity was detected only in lung samples from aborted fetuses. The strain was successfully isolated using MDBK cells and was named LC1. After five passages, the isolate demonstrated stable cytopathic effects, such as nuclear condensation and cytoplasmic fragmentation when cultured for 48 h (Figure 1A). No CPE was observed in the negative control culture (Figure 1B). The virus titer was 10^7.5^TCID_50_/mL. Indirect immunofluorescence assays showed strong BPIV3 antigen specific fluorescence in the cytoplasm (Figure 1C), and electron transmission electron microscopy revealed that the isolate had a diameter of approximately 250 nm and vesicle membranes (Figure 1D). The HA titer of the LC1 strain was 1:8. After primer amplification, we obtained a complete genome sequence of 15,474 bp in length, containing all coding regions for six structural proteins (N, P, L, M, HN, and F) and three non-structural proteins (C, V, and D). Comparative genomic analysis revealed that the number, transcriptional orientation, and sequential arrangement of genes were highly conserved among BPIV3 strains, consistent with the typical genomic organization of BPIV3 genotype C (3′-N-P(C/V/D)-M-F-HN-L-5′). This sequence has been uploaded in the GenBank database under the accession number PQ074215.

**Figure 2:**
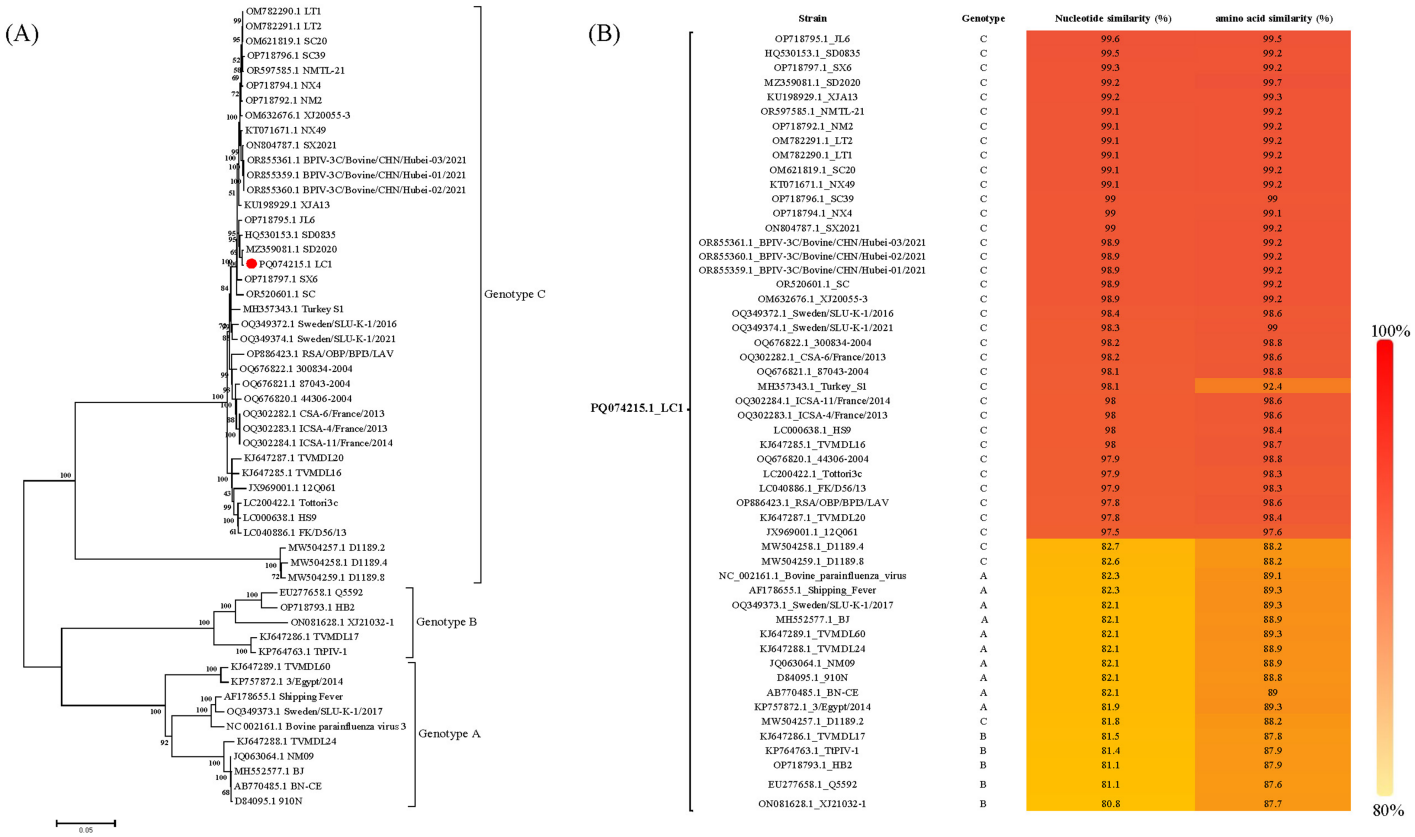
Sequences analysis of BPIV3 strains was conducted based on the all nearly complete genome sequences. **(A)** The best-fit substitution model for the phylogenetic tree constructed from the nearly complete coding genome, was determined to be the general time-reversible model with gamma distribution and invariant sites (GTR + G + I). The sequences were analyzed using the maximum likelihood method with 1,000 bootstrap replicates. The 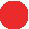 symbol represents the abortion-derived BPIV3 sequences identified in this study. **(B)** Nucleotide and amino acid similarity between LC1 and all genotypes of BPIV3.

Phylogenetic analysis based on the complete genome revealed that this isolate belonged to genotype C and clustered into a branch with Chinese BPIV3 genotype C strain SD2020 (Figure 2A). The complete genome of the LC1 isolate showed nucleotide similarity ranging from 80.8 to 99.6% and complete coding sequences of all viral proteins showed amino acid similarity ranging from 87.6 to 99.7% compared to BPIV3 strains representing all genotypes in the GenBank database; the nucleotide similarity of LC1 to all BPIV3 C strains in the GenBank database was 81.8 to 99.6%, and complete coding sequences of all viral proteins showed amino acid similarity was 88.2 to 99.7%, showing the highest amino acid homology to the BPIV3 genotype C strain SD2020, which was isolated in Shandong Province, China (Figure 2B). The recombination analyses revealed no recombination events for this genome.

**Figure 3:**
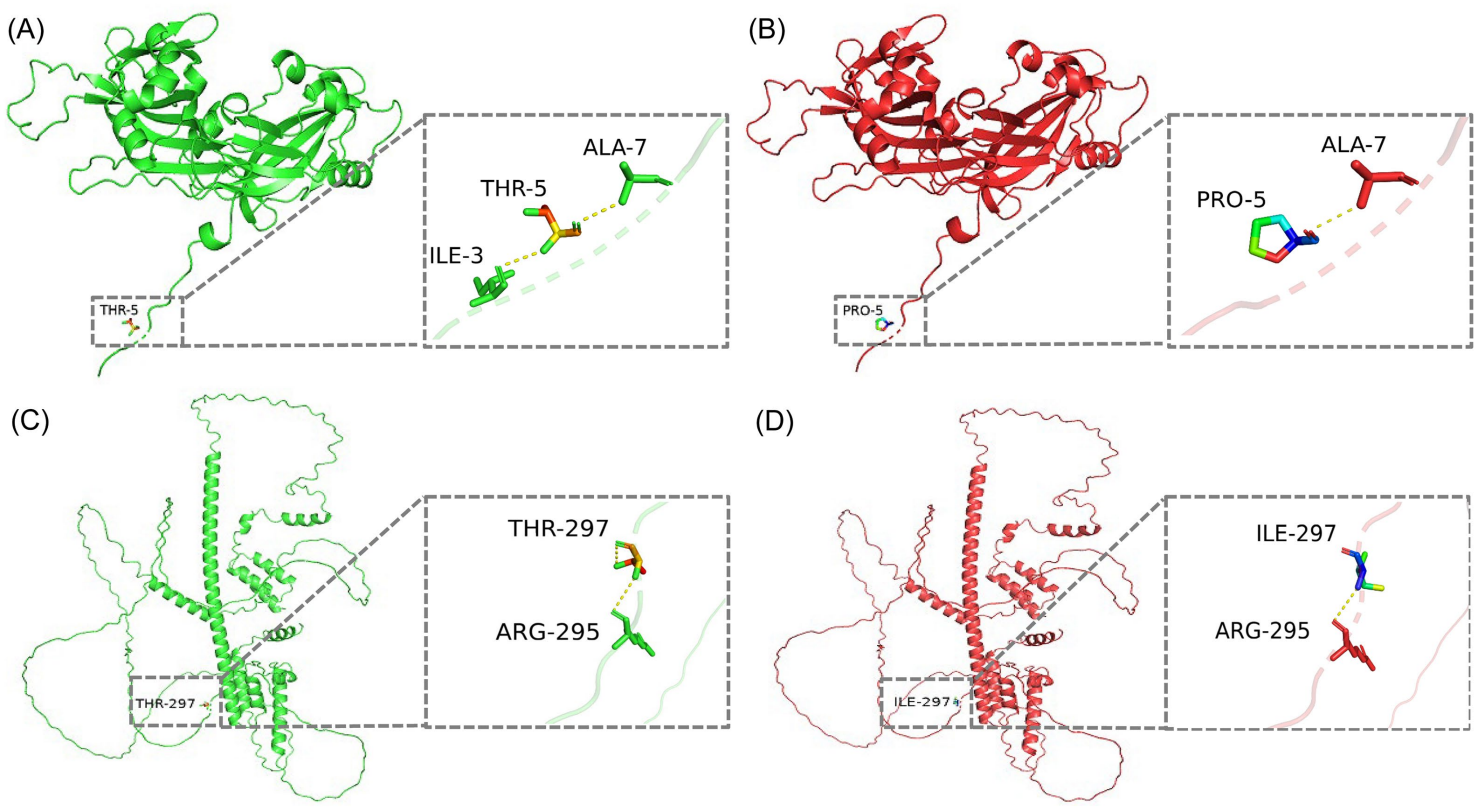
3D conformational maps of P and M proteins. **(A)** Conformation of M protein of SD2020 reference strain. **(B)** Conformation of M protein of the isolate of this study. **(C)** Conformation of P protein of SD2020 reference strain. **(D)** Conformation of P protein of the isolate of this study. In each conformation, the enlarged boxes highlight the mutation sites, and the yellow dashed lines represent hydrogen bonds.

Complete-genome sequence analysis is a powerful tool for studying pathogenicity, pathogenic mechanisms, and transmission routes of the pathogen. Further analysis of all BPIV3 genotype C complete genome sequences available in the GenBank database revealed a total of six unique nucleotide mutations in its *P* (T890C, T1191C), M (C13A), and *L* (C483T, G5046A, C6183T) genes. Two of these mutations were sense mutations, resulting in unique changes at position 297 for the P (I297T) protein, and at position 5 for the M (P5N) protein. Modeling of P and M proteins and subsequent analysis after introducing mutations showed that the amino acid mutations reduced the number of hydrogen bond (H-bond) in the P and M proteins by one at these sites (Figure 3).

Selection pressure analyses of the BPIV3 *N*, *P*, *M*, *F*, *HN*, and *L* genes revealed that the dN/dS values varied across genes: the *N* gene ranged from 0 to 0.1133, the *P* gene from 0.005 to 0.595, the *M* gene from 0.006 to 0.624, the *F* gene from 0 to 0.5906, the *HN* gene from 0 to 0.521, and the *L* gene from 0.031 to 0.227. Notably, the dN/dS values for all genes were significantly less than 1, suggesting that these genes are predominantly subjected to negative selection pressure. Further analysis of mutations at unique amino acid sites in the P (I297T) and M (P5N) proteins indicated that mutations in the P protein were predicted to experience positive selection pressure by three algorithms (MEM, FEL, and FuBAR). In contrast, the mutation in the M protein was predicted to be under positive selection pressure by only the MEM algorithm.

## Discussion

BPIV3 is one of the pathogens causing reproductive disorders in cattle (15), but the research data on BPIV3 related to abortion in dams remain scarce. Previous research has demonstrated that BPIV3 genotype A strains have been identified in aborted Holstein fetuses in the United States, causing tissue damage, including necrotizing bronchiolitis/alveolitis, intrabronchial/alveolar fibrin exudation, non-suppurative-peribronchitis, perivascular interstitial pneumonia, and necrosis of intestinal epithelial cells (13). BPIV3 genotype B strains were isolated from vaginal swabs of buffaloes with reproductive diseases in Argentina (10), implying that BPIV3 can be transmitted through the reproductive tract. Given the close anatomical connection between the vagina and the uterus, there is a potential for the virus to be transmitted to the uterus through retrograde infection, which could subsequently impact fetal health. However, to our knowledge, this study is the first to report the isolation of a genotype C strain from an abortion sample. Therefore, further research is needed on the pathogenicity, pathogenic mechanisms, and transmission routes of BPIV3 genotype C strains in the bovine reproductive system. In addition, we amplified the nearly complete genome of the abortion-derived BPIV3 and uploaded it to the GenBank database. The release of this genome sequence fills a gap in the existing data and lays a foundation for further molecular biology research and exploration of the pathogenic mechanism.

Phylogenetic analysis confirmed that the LC1 isolate belongs to BPIV3 genotype C, forming a closely related clade with SD2020 (GenBank accession number MZ359081.1), a respiratory-derived strain from China. This genetic relationship is supported by high nucleotide and amino acid sequence identities between LC1 and SD2020, despite their isolation from geographically distant provinces, Heilongjiang and Shandong, respectively, which are separated by over 1,000 km in a straight line. These findings underscore the necessity of comprehensive, regionally inclusive genomic surveillance of BPIV3 to effectively track viral evolution and transmission dynamics. However, further sequence analysis revealed that the abortion-derived BPIV3 genome had four synonymous mutations in the *P* and *L* genes, as well as one missense mutation each in the P (I297T) and M (P5N) proteins. These missense mutations resulted in the loss of an H-bond at the mutation sites in the P and M proteins, respectively. H-bonds play a critical role in biochemical reactions, such as protein folding, protein-ligand, protein-lectin, or protein–protein interactions, which may lead to an alteration in their conformation and function (22). In addition, synonymous codon substitutions are known to control the level of ribosomally expressed proteins affecting protein folding (23). Therefore, both synonymous and missense mutations may have important biological significance, and further analyses are needed to determine the functional characterization of these mutations. Notably, the genomic information for the strain we studied was obtained from an abortive sample, representing only genomic data from an abortive source, while all other known BPIV3 genomic comes from respiratory samples. This unique source emphasizes the possibility that this mutant strain may possess specific biological characteristics associated with abortion. More genomic sequences from aborted isolates will be needed in the future to identify this unique mutation.

Selection pressure plays a crucial role in viral evolution, influencing genetic diversity and adaptability (24). The selection pressure analysis of BPIV3 genes (N, P, M, F, HN, and L) provides valuable insights into the evolutionary dynamics of this virus. The results of this study indicate that all the examined BPIV3 genes are under negative selection pressure, which is essential for eliminating deleterious mutations and maintaining the functional integrity of key viral proteins. Additionally, predictive analyses of unique amino acid mutation sites in the P (I297T) and M (P5N) proteins revealed that localized sites may experience positive selective pressure. This suggests that these mutations could confer adaptive advantages, aiding the virus in adjusting to host environments or enhancing its transmission capabilities. Collectively, these findings underscore the intricate interplay of selection pressures in shaping BPIV3 evolution.

Currently, research on the P and M proteins of BPIV3 strains is limited. Existing studies indicate that the open reading frame of the *P* gene not only translates the phosphoprotein but also encodes three non-structural proteins (V/C/D), which primarily function to inhibit host cell interferon production (25). The M protein plays a critical role in virus assembly, budding, and release (26). A recent study revealed that the first 10 amino acids at the N-terminus and the last 4 amino acids at the C-terminus of the BPIV3 M protein are crucial for the release of viral particles (27). In this study, the isolate shows a mutation at the fifth amino acid at the N-terminus of the M protein. Therefore, we hypothesize that this mutation could potentially affect the release of viral particles. This potential impact on viral release merits further investigation to understand the biological consequences associated with this mutation. In conclusion, the unique amino acid mutations identified in the P and M proteins of the isolates in this study necessitate further investigation to elucidate their impact on the biological properties of these strains.

## Conclusion

In this study, we successfully isolated an abortion-derived BPIV3 genotype C strain from the aborted fetus of a Holstein sample and obtained the nearly complete genome. This work confirms the existence of an abortion-derived BPIV3 genotype C strain and provides valuable data for its molecular characterization. Complete genome sequence analysis identified unique amino acid mutations in the P and M proteins of the isolate. These mutations may significantly impact the biological properties of the isolate; therefore, future research should focus on elucidating the specific effects of these mutations on the virus’s biological characteristics and their role in the BRDC and reproductive disorders.

## Data Availability

The data presented in this study can be found in the NCBI database, accession numbers: PQ074215.

## Footnotes

^1^ https://cosmic-cryoem.org/tools/alphafold/

^2^ www.datamonkey.org
